# From the Lalonde Report to the structural determinants of health

**DOI:** 10.17269/s41997-025-01070-0

**Published:** 2026-01-05

**Authors:** Katherine L. Frohlich

**Affiliations:** Institute of Population and Public Health, Canadian Institutes of Health Research, Ottawa, ON Canada

**Keywords:** Social determinants, Structural determinants, Health promotion, Equity, Lalonde, Déterminants sociaux, Déterminants structurels, Promotion de la santé, Équité, Lalonde

## Abstract

**Abstract:**

In this commentary, I discuss the history and legacy of Marc Lalonde’s ground-breaking report on health promotion and critically assess how far we have come in building a society that produces health, not sickness. In doing so, I will share the arc that I see between the Lalonde Report and our new Strategic Initiative at CIHR’s Institute of Population and Population Health (IPPH), Moving Upstream, which focuses on the structural determinants of health. I propose that Moving Upstream can help us move the needle on equitable health promotion research, policy, and practice in Canada, thus building on the foundation created by Lalonde.

**Résumé:**

Dans ce commentaire, j'aborde l'histoire et l'héritage du rapport de Marc Lalonde sur la promotion de la santé, et j'évalue de manière critique les progrès accomplis dans l'édification d'une société axée sur la santé, et non sur la maladie. Ce faisant, je présenterai le lien que je vois entre le rapport Lalonde et notre nouvelle initiative stratégique à l'Institut de la santé et des populations (ISPP) des IRSC, «Agir en Amont», qui développe la recherche sur les déterminants structurels de la santé. Je propose que «Agir en Amont» puisse nous aider à faire progresser la recherche, les politiques et les pratiques en matière de promotion de la santé au Canada, en nous appuyant sur les bases posées par Lalonde.

The celebration held at McGill University in April 2024 asked participants to reflect on how we have collectively moved the needle on equitable health promotion since the publishing of the Lalonde Report (Lalonde, 1974), 50 years earlier. In this commentary, I discuss the history and legacy of Marc Lalonde’s ground-breaking report on health promotion and critically assess how far we have come in building a society that produces health, not sickness. In doing so, I will share the arc that I see between the Lalonde Report and our new Strategic Initiative at CIHR’s Institute of Population and Population Health (IPPH), *Moving Upstream*, which focuses on the structural determinants of health. I propose that *Moving Upstream* can help us move the needle on equitable health promotion research, policy, and practice in Canada —thus building on the foundation created by Lalonde.

As this special issue fulsomely demonstrates, the Lalonde Report was innovative in the envisioning of public health/health promotion in a number of ways. The most transformative of the additions to public health, for the sake of the argument developed here, was his report’s description of, and hierarchy given to, “the four broad elements of the health field”, or what are now known as the determinants of health. In his list of four broad elements, Lalonde positioned health care organization, or the medical care system, last in order of importance. This was deliberate. But the report did not just deprioritize medical care. It also shone light on new factors, specifically what he called lifestyle factors and the social environment. These areas of focus hailed a new era of thinking concerning factors that individuals and populations have some level of agency over: radically different from others which are, as he calls them, “out of our control”.

Of course, there is continuing significant debate in public health regarding just how much agency populations have over the health “choices” they make, but suffice it to say, Lalonde officially began this debate by placing these two factors as being significant in shaping health and disease. I would argue, indeed, that our thinking about how “out of individual control” these lifestyle and environmental factors are has shifted considerably over the past 50 years. And these shifts, which I will discuss later, have paved the path for us to begin serious engagement within the Canadian public health research, practice, and policy realms around the structural determinants of health.

Fast forward some 12 years to 1986. WHO Europe organizes its conference in Ottawa and the result is what we now call the Ottawa Charter for Health Promotion. Unlike the Lalonde Report, which focused on a definition of the health field, or a way of defining what “causes health and disease”, the Ottawa Charter turned attention to the *how* of health promotion and public health: the various means that health can be created and improved.

The Ottawa Charter laid out five key action areas in health promotion, or axes: building healthy public policy, creating supportive environments for health, strengthening community action for health, developing personal skills, and re-orienting health services. The Charter also recommended three basic health promotion strategies to guide change (to enable, mediate, and advocate).

While for those of us steeped in the health promotion tradition, the Ottawa Charter is a sort of original document; I would maintain that it is too frequently omitted from conversations about where we need to go in public health and how. I would draw particular attention to the action area of the Ottawa Charter called “Building Healthy Public Policy”. This area was innovative at the time for moving beyond what had formerly been known as public health policy: a policy that was largely concerned with health problems based on a biomedical determinants of health model (Holt & Frohlich, 2022). Healthy public policy, differently from public health policy, was developed in the Charter as being concerned with the conditions that create a healthy and equal society based on the social determinants of health model. This axis was clearly the predecessor to what we now call Health in all Policies, a framework that undergirds our new *Moving Upstream* strategic initiative.

Fast forward again some 22 years. Sir Michael Marmot becomes the Chair of the WHO Commission on the Social Determinants of Health (CSDH). This Commission was established to support countries and global health partners to address the social factors leading to ill health and inequities. It drew the world’s attention to the social determinants of health known to be among the worst causes of poor health and inequalities between and within countries. The report that resulted from this Commission (WHO, 2008) yielded three overarching recommendations:

1. Improve daily living conditions.
2. Tackle the inequitable distribution of power, money, and resources.
3. Measure and understand the problem and assess the impact of action.

This was the first report of its global status to explicitly call out the unequal distribution of power, money, and resources as key system drivers that maintain health inequity. Moreover, they named this as a political endeavour.

The report also importantly drew the direct link between inequities in conditions of people’s lives—their access to health care, schools, education, and housing—and their chances to reach their full health potential. This distinctly intersectoral approach to overcoming inequities opened up new ways of thinking about theories, methods, approaches, and policies to tackle health inequities.

When the CSDH published their attendant framework on the social determinants of health (SDOH) (Solar & Irwin, 2010), they intentionally distinguished between the mechanisms by which social hierarchies are created and the conditions of daily life which then result. WHO’s definition continues to include both “the conditions in which people are born, grow, live, and age” and “the wider set of forces and systems shaping the conditions of daily life”. Since the CSDH framework publication, however, it has commonly been operationalized as the conditions of daily life alone, obfuscating the more “upstream” determinants creating these conditions. This limited use of the framework depoliticizes the concept of the SDOH, perhaps a result of dominant neoliberal worldviews (Heller et al., 2024).

What does this mean for the science around these “upstream”, more structural factors? Up until now, I think we have done quite well in health promotion, social epidemiology, and public health in general on the side of what some call “the science of problems”. We are extremely good at demonstrating empirically that social inequities in health exist. Again and again, across social determinants, we demonstrate inequities in morbidity and mortality for a wide variety of diseases and risk factors. What we are much less good at thinking through and demonstrating is what we can actually do to reduce these inequities or to fulsomely engage in what is called the science of solutions.

So, what can we do differently? First, clearly there is a need for more and different types of interdisciplinary work. These upstream questions are not for epidemiologists or health promoters alone. We need to work alongside political scientists, economists, policy experts, anthropologists, urban planners, and the list goes on.

Second, now even more than ever we need to think inter-sectorally. We know that health promotion and public health need to work more with public, non-profit, and private organizations in spaces that do not fall directly into public health: organizations working on questions of the environment, food production, education, and law. Recent debates on the commercial determinants of health, for instance, bring a new perspective to the duality of structure and agency; global corporations develop new products, market, engage in research, and enact legislation regarding myriad health-related social practices (tobacco, ultra-processed foods) (Diderichsen et al., 2021), shaping individual agency in new ways.

And the science of solutions can come about in changes at every level, given that health is produced in people’s homes, neighbourhoods, cities or towns, provinces, countries, and the planet. The drivers of health are potentially different at these various levels, but they certainly exist and are mutable.

We have developed a new area of research, policy, and practice investment at the Institute of Population and Public Health which has as its goal to nurture a robust range of interdisciplinary research approaches that seek to understand the way structural determinants create, maintain, and exacerbate health (in)equities in Canada. We anticipate that the research resulting from this investment will help build on Lalonde’s legacy, moving us towards new understandings of the “health field” and opportunities to attain more equitable health outcomes for Canadians.

## Data Availability

Not applicable.
